# Activation of 5-HT7 receptor stimulates neurite elongation through mTOR, Cdc42 and actin filaments dynamics

**DOI:** 10.3389/fnbeh.2015.00062

**Published:** 2015-03-11

**Authors:** Luisa Speranza, Teresa Giuliano, Floriana Volpicelli, M. Egle De Stefano, Loredana Lombardi, Angela Chambery, Enza Lacivita, Marcello Leopoldo, Gian C. Bellenchi, Umberto di Porzio, Marianna Crispino, Carla Perrone-Capano

**Affiliations:** ^1^Department of Biology, University of Naples Federico IINaples, Italy; ^2^Institute of Genetics and Biophysics “Adriano Buzzati Traverso”, CNRNaples, Italy; ^3^Department of Pharmacy, University of Naples Federico IINaples, Italy; ^4^Department of Biology and Biotechnology “Charles Darwin”, Istituto Pasteur Fondazione Cenci Bolognetti, University of Rome La SapienzaRome, Italy; ^5^Department of Environmental, Biological and Pharmaceutical Sciences and Technologies, Second University of NaplesNaples, Italy; ^6^IRCCS, MultimedicaMilano, Italy; ^7^Department of Pharmacy - Pharmaceutical Sciences, University of BariBari, Italy

**Keywords:** 5-HT7 receptor, actin dynamics, axonal elongation, Cdc42, mTOR, neurite outgrowth

## Abstract

Recent studies have indicated that the serotonin receptor subtype 7 (5-HT7R) plays a crucial role in shaping neuronal morphology during embryonic and early postnatal life. Here we show that pharmacological stimulation of 5-HT7R using a highly selective agonist, LP-211, enhances neurite outgrowth in neuronal primary cultures from the cortex, hippocampus and striatal complex of embryonic mouse brain, through multiple signal transduction pathways. All these signaling systems, involving mTOR, the Rho GTPase Cdc42, Cdk5, and ERK, are known to converge on the reorganization of cytoskeletal proteins that subserve neurite outgrowth. Indeed, our data indicate that neurite elongation stimulated by 5-HT7R is modulated by drugs affecting actin polymerization. In addition, we show, by 2D Western blot analyses, that treatment of neuronal cultures with LP-211 alters the expression profile of cofilin, an actin binding protein involved in microfilaments dynamics. Furthermore, by using microfluidic chambers that physically separate axons from the soma and dendrites, we demonstrate that agonist-dependent activation of 5-HT7R stimulates axonal elongation. Our results identify for the first time several signal transduction pathways, activated by stimulation of 5-HT7R, that converge to promote cytoskeleton reorganization and consequent modulation of axonal elongation. Therefore, the activation of 5-HT7R might represent one of the key elements regulating CNS connectivity and plasticity during development.

## Introduction

The neurotransmitter serotonin (5-hydroxytryptamine, 5-HT) modulates a variety of physiological processes in the Central Nervous System (CNS). In addition to its well-established role in neurotransmission, 5-HT has been shown to regulate the connectivity of the brain by modulating cellular migration and cytoarchitecture during development (Daubert and Condron, 2010). Mammalian brain contains extensive serotonergic projections that exert their effects through at least 14 different subtypes of 5-HT receptors, classified into 7 different families (Pytliak et al., 2011). The 5-HT receptor 7 (5-HT7R) is the most recently identified 5-HT receptor. This seven-transmembrane G-protein-coupled receptor is positively linked to adenylate cyclase through the stimulatory Gs protein and Gα-12 subunit of heterotrimeric G-protein (Kvachnina et al., 2005; Leopoldo et al., 2011), and it is expressed in CNS and periphery. In human and rodent CNS, high densities of 5-HT7R have been found in hypothalamus, thalamus, hippocampus, cortex, amygdala and striatal complex. This distribution is highly correlated with 5-HT7R functions in the CNS, namely circadian rhythm, REM sleep, thermoregulation, learning and memory and nociception (Matthys et al., 2011; Adriani et al., 2012). Consistent with such a broad range of functions, its deregulation has been associated with numerous pathological processes of the CNS, such as obsessive**—**compulsive disorder (OCD), anxiety, schizophrenia, epilepsy, migraine, sensation-seeking behavior, impulsivity and depression (Cates et al., 2013; Gellynck et al., 2013).

Although precise biological roles 5-HT7R are not well known yet, recent studies suggest that this receptor might be implicated in the modulation of synaptic wiring in the CNS. Interestingly, 5-HT7R expression was markedly increased in adolescent rodents administered with methylphenidate. These animals display specific changes in brain reward circuits and in the reward-based behavior, suggesting that 5-HT7R may play a major role in the modulation of self-control behavior by supporting the persistent brain structural rearrangements during postnatal development (Adriani et al., 2006; Leo et al., 2009). Indeed, it has been shown that 5-HT7R modulates hippocampal neuronal morphology, excitability and plasticity, by participating in dendritic morphogenesis and synaptogenesis (Kobe et al., 2012; Tajiri et al., 2012). In line with these results, pharmacological stimulation of 5-HT7R with a new selective agonist, LP-211 (Hedlund et al., 2010), has been successfully employed to rescue the synaptic plasticity deficits in hippocampal slices from a mouse model of the X-fragile syndrome (Costa et al., 2012) and to alleviate the behavioral phenotype in a mouse model of the Rett syndrome (De Filippis et al., 2014). Thus, all these recent findings indicate that 5-HT7R controls neuritogenesis and synaptogenesis, but the signaling mechanisms remain poorly understood.

Recently we have shown that 5-HT7R activation in neuronal primary cultures from rat brain stimulates neurite outgrowth through Cdk5 and extracellular signal-regulated kinases 1/2 (ERK) pathways, with the modifications of selected cytoskeletal proteins (Speranza et al., 2013), supporting the hypothesis that the 5-HT7R might play a crucial role in shaping neuronal networks during development (Volpicelli et al., 2014). The modulation of connectivity in the nervous system requires remodeling of neuronal morphology and synaptic connections, which in turn depend on dynamic reorganization of cytoskeleton. Various extracellular cues induce changes of the actin cytoskeleton to remodel the structure and the function of subcellular regions by way of numerous signaling pathways, and dynamic changes of actin filaments are crucial for neurite outgrowth and axonal pathfinding and synaptogenesis (Schaefer et al., 2008). One of the most characterized actin-binding proteins, cofilin, binds, twists and severs actin filaments enabling remodeling of the actin cytoskeleton (Bernstein and Bamburg, 2010; Bravo-Cordero et al., 2013). Cofilin is regulated by the upstream signaling pathways involving Rho/Rac/Cdc42 GTPase; in turn, the Rho signaling pathway and the consequent cofilin-dependent actin polymerization are, in many cells, under the control of the mammalian target of rapamycin (mTOR; Jaffe and Hall, 2005; He et al., 2013). The latter is a multifunctional serine/threonine protein kinase which also plays important roles in neurite elongation, dendritic spines shape and synaptogenesis (Guo et al., 2011; Briz and Baudry, 2014; Takei and Nawa, 2014).

In this work we have first demonstrated that the stimulation of 5-HT7R with the selective agonist LP-211 (Hedlund et al., 2010) leads to enhanced neurite outgrowth in embryonic neuronal primary cultures derived from the cortex (CTX), hippocampus (HIPP) and striatal complex (STR) of embryonic mouse brain corroborating the results that we have previously obtained in rat brain (Speranza et al., 2013). As an extension of this finding, we have herein analyzed several intracellular transduction pathways connecting the stimulation of 5-HT7R to neurite elongation. We have found that, in addition to ERK phosphorylation and Cdk5 activation, mTOR and Cdc42 are activated during the 5-HT7R-dependent neurite elongation. With all these pathways converging in the modulation of the dynamics of neuronal cytoskeleton proteins, our data indicate that signals activating 5-HT7R is transduced to neurite elongation via remodeling of actin cytoskeleton. In addition, by use of the microfluidic chambers, we have demonstrated for the first time that the neurites outgrowing in response to 5-HT7R stimulation include axons, suggesting an unexpected role of this receptor in axonal pathfinding and regeneration.

## Materials and methods

### Neuronal primary cultures

Timed pregnant C57 BL/6 mice were housed, cared for and sacrificed in accordance with the recommendations of the European Commission. All the procedures related to animal treatments were approved by Ethic-Scientific Committee for Animal Experiments and Italian Health Ministry. The embryonic age (E) was determined by the date of insemination (i.e., the appearance of vaginal plug was considered as day E0). For every cell culture preparation, about 15–20 embryos from different dams were pooled. The STR and CTX from E15 embryos, and the HIPP from E18 embryos were dissected under a stereoscope in sterile conditions, and placed in PBS without calcium and magnesium, supplemented with 33 mM glucose. Cells dissociated from embryonic STR, CTX, and HIPP were cultured as previously described (di Porzio et al., 1980). Briefly, the dissected areas were enzymatically dissociated by incubation for 30 min at 37°C in a papain solution (Warthington, 20 U/ml, Milan, Italy) in Earle's balance salts containing 1 mM EDTA (Sigma-Aldrich, Milan, Italy), 1 mM cysteine (Sigma-Aldrich) and 0.01% pancreatic DNAse (Sigma-Aldrich). After addition of 1 mg/ml of bovine serum albumin (Sigma-Aldrich) and 1 mg/ml ovomucoid (Sigma-Aldrich) the cells suspensions were centrifuged 5 min at 800 × g, resuspended in plating medium and counted (Fiszman et al., 1991). For the viable cell count, cell suspension was diluted 1:1 with 0.1% trypan blue dye (Sigma-Aldrich) and loaded into a disposable cell counting chamber-slide. Cell concentration was determined on the basis of the total cell count, the dilution factor and the trypan blue dye exclusion.

Dissociated cells were plated at a density of 1.5 × 10^5^/cm^2^ in 2 cm^2^ Lab-Tek chamber slides (Nunc) for morphological analyses, and at a density of 3 × 10^5^/cm^2^ in 9,5 cm^2^ cell culture dishes (Corning) for RNA purification and Western blot analyses. Both chamber slides and cell culture dishes were coated with 15 μg/ml of poly-D-Lysine dissolved in water (Sigma-Aldrich).

Cultures were grown in serum-free Neurobasal medium (Life technologies, Milan, Italy), supplemented with B27 (Life technologies), 2 mM L-glutamine (Sigma-Aldrich), penicillin (50 U/ml, Sigma-Aldrich) and streptomycin (50 μg/ml, Sigma-Aldrich). Cells were maintained for 3 days *in vitro* (DIV) at 37°C in a humidified incubator in presence of 5% CO_2_, before experimental manipulation. For each experimental point, cultures were prepared at least in independent triplicates, and were repeated using distinct culturing sessions.

### Drugs and reagents

The cell cultures were treated with 100 nM of the selective 5-HT7R agonist LP-211 (gift of M. Leopoldo, University of Bari, Italy), 100 nM of the HT7R antagonist SB-269970 (Tocris, Milan, Italy; Hagan et al., 2000), or with a combination of these drugs. Roscovitine (Sigma-Aldrich), a Cdk5 inhibitor, was used at the final concentration of 20 μM. The mTOR inhibitors rapamycin (Sigma-Aldrich) and torin 1 (Tocris), were used at a final concentration of 20 and 250 nM, respectively. ZCL 278 (Tocris), a selective inhibitor of Cdc42, was used at a final concentration of 50 μ M. Cytochalasin D (Sigma -Aldrich) was used at a final concentration of 100 nM, while latrunculin and jasplakinolide (Molecular Probes, Milan, Italy) were used at a final concentration of 2 μ M. Cells were pretreated for 30 min with 10 μM of U0126, the ERK 1/2 inhibitor, as recommended by manufacturer (Cell Signaling, Milan, Italy). Drugs were added to cultures 72 h after cell plating and incubated for appropriate time.

### RNA isolation and RT-PCR analyses

Total RNA was extracted from primary cells cultured in 4 cm^2^ wells, 3 days after seeding, using the Tri-Reagent according to the manufacturer's instructions (Sigma-Aldrich). The analyses were always carried out in triplicate samples for each experimental point. Reverse transcriptase and quantitative real time PCR analyses were performed as described in Speranza et al. (2013). Primer sets used were:

5-HT7R: Fw GCGGTCATGCCTTTCGTTAGT—Rev GGCGATGAAGACGTTGCAG;

HPRT: Fw TGGGAGGCCATCACATTGT—Rev AATCCAGCAGGTCAGCAAAGA.

Gene expression levels were quantified by the comparative threshold cycle (*CT*) method (Schmittgen and Livak, 2008) using hypoxanthine phosphoribosyltransferase (HPRT) as an internal control gene. The fractional number of PCR cycles (*CT*) required to obtain a given amount of amplified product in the exponential phase of amplification was determined for the gene of interest and for HPRT in each cDNA sample. The relative expression level of the gene of interest was then expressed as 2^−Δ*CT*^ where Δ*CT* = *CT* gene of interest—*CT* HPRT.

### Morphological characterization and analysis of morphometric parameters

For morphological characterization of neuronal cultures, cells were fixed in 4% paraformaldehyde in phosphate buffered saline (PBS), for 30 min at room temperature (RT), washed three times in PBS, and then permeabilized for 20 min in PBS containing 0.1% Triton-X-100 and 10% normal goat serum (NGS). Cells were treated with blocking solution [10% NGS, 0, 1% bovine serum albumine (BSA) in PBS] at RT for 1 h and incubated with the primary antibody in antibody solution (0, 1% BSA in PBS) overnight at 4°C. The following antibodies were used at the indicated dilutions: monoclonal antibody against neuron specific class III β-tubulin (Tuj1, Covance, Milan, Italy) 1:500 and polyclonal antibody 5-HT7 receptor (Imgenex, Milan, Italy) 1:70. The cells were washed in PBS, and then incubated with fluorescent secondary antibodies (Alexa Fluor goat anti-rabbit, and Alexa Fluor Goat goat anti-mouse, Life technologies) diluted 1:400 in antibody solution.

Cells were then counterstained with DAPI (nuclear stain, 1:1000) for 10 min, washed with PBS and mounted with oil mounting solution (Mowiol). Fluorescent signals from Tuj1 stained neurons were detected with a microscope (Leica DM6000B) equipped with an objective 20x. Images were acquired with high-resolution camera using the software Leica Application Suite, and were analyzed by the image-processing software Image J, for the perimeter and the area of the soma, neurites number and length. In each image, the neuronal cells were recognized by their immunoreactivity with Tuj1 antibody. Using the Image J software the images were pre-processed to optimize illumination and contrast. The length of the neurites was estimated by measuring the length of a line manually drawn from the soma to the end of the primary neurites (neurites that originate directly from soma) using the “Measure” function of the software (modified from Hannan et al., 2014; **Figure 2A**).

Only clearly visible cells were subjected to analysis to prevent inaccurate scoring. A total of 15 fields for each cell-culture condition was selected from at least three independently treated culture wells. A total of 300 neurites/well was traced from Tuj1 positive neurons to measure their length. The analyses were carried out blindly to avoid any subjective influences during the measurements.

Morphometric parameters were always compared to the controls from the same batch of dissociated cells treated with vehicle alone for the same time length (CTRL). Significant agonist-induced increase in neurite length varied always between 1.2 and 2 fold compared to CTRL. For easy comparison of the results among various cell preparations, data were expressed as percentage of the average CTRL.

### Incubation and immunofluorescence in microfluidic chambers

Hippocampal neuron cultures were prepared from 18-day old (E18) mouse embryos. The hyppocampi were dissected in cold HBSS 1X and 3 mM HEPES (Life Technologies), containing antibiotics (100 unit/ml penicillin and 0.1 mg/ml streptomycin, Sigma-Aldrich), and transferred into a 15 ml tube. After a rinse in buffer, specimens were incubated, for 30 min at 37°C, with 40 μ l/HIPP of 0.5% trypsin (EuroClone SpA, Pero, Italy) (equivalent to 0.2 mg trypsin/HIPP) and 100 μg/ml DNAase. After an extensive wash, hippocampi were mechanically dissociated and cell density was determined using a counting chamber. Cells were re-suspended in 100 μ l of the appropriate medium and plated in microfluidic chambers (Xona Microfluidics LLC, Temecula, California USA; Taylor et al., 2005; Park et al., 2006). Chambers were prepared as suggested by manufacturer's instructions: briefly, 35 mm Petri dishes were coated overnight with 100 μg/ml poly-L-lysine (Sigma-Aldrich) at 37°C and then washed and air dried under a sterile hood. Poly-dimethylsiloxane chambers were placed on the Petri dishes with their micro-channel side down, sealed to the dish by gentle pressure and filled with 20 μg/ml laminin (Sigma-Aldrich), for 2 h at 37°C. Laminin was removed just before plating cells, approximately 100,000 cells were pipetted directly into one compartment (the soma compartment) and then both compartments were filled with Neurobasal medium containing 1X B27 supplement (Life Technologies), 0.5 mM glutamine, 25 μ M glutamate and antibiotics. When neurons were plated at this density, very few cells approached the micro-channels, so that most likely one axon/channel enters. Cells were exposed to DMSO (control) or DMSO + LP-211, added to both compartments (somatic and axonal), and maintained *in vitro* for 6 days (6 DIV). Medium was changed every 24 h and the number of axons reaching the axonal compartment was blindly counted every day by three operators, from 1 to 6 DIV, at the inverted microscope, in phase contrast, at a magnification of 40x. Finally, at 6 DIV, after the last count, cells were fixed in 4% formaline and 4% sucrose in PBS for 30 min at RT, added directly to both compartments, without removing the microfluidic chambers. After fixation, cells were blocked in 1% BSA (Sigma-Aldrich), 10% normal goat serum (NGS) (Jackson ImmunoResearch Europe Ltd, Suffolk, England) and 0.5% Triton X-100 in PBS, for 1 h at RT, and then incubated overnight at 4°C with the primary antibodies diluted in 1% bovine serum albumin, 1% NGS and 0.2% Triton X-100 in PBS. Antibodies used were: mouse anti-neuron specific βIII-tubulin (Tuj1; Covance, Emeryville, CA, USA, #MMS-435P; 1:3000), in co-localization with either rabbit anti Map2 (AbCam, Cambridge, UK, #32454; 1:400), to visualize dendrites, or rabbit anti Tau (AbCam, #ab64193; 1:50), to visualize axons. Cells were then incubated for 1 h at RT with the appropriate secondary antibodies: goat anti-mouse IgG Alexa Fluor 488 (Molecular Probes, Life Technologies) and goat anti-rabbit IgG Cy3 (Jackson ImmunoResearch), both diluted 1:1000. ProLong Gold Antifade Reagent (Invitrogen, Life Technologies) was added to both compartments to visualize and store immunolabed cells.

### Gel electrophoresis and western blot analyses

For mono-dimensional polyacrylamide gel electrophoresis (PAGE), culture dishes were lysed in RIPA Buffer in presence of protease inhibitors (Roche, Milan, Italy). Proteins (15–30 μg/lane) were separated on 10–12% SDS-polyacrilamide gel and transferred to PVDF membranes (GE Healthcare, Milan, Italy). For experiments with S6 kinase, Akt and cofilin antibodies, culture dishes were lysed in 20 mM MOPS pH 7, 2 mM EGTA, 5 mM EDTA, 30 mM NaF, 60 mM β-glycerol-phosphate, 1 μM sodium orthovanadate, 1% triton), in presence of protease inhibitors. Proteins (20–30 μg/lane) were separated as previously described and transferred to PVDF membranes. Filters were blocked for 30 min in 5% (w/v) non-fat milk in Tris-buffered saline Tween-20 (TBST; 0.1% Tween, 150 mM NaCl, 10 mM Tris-HCl, pH 7.5) and probed for 2 h at RT with the following antibodies: anti-5-HT7 receptor (Imgenex IMG-368, 1:300), anti-β-actin (BD Transduction Laboratories, #612656, 1:1000), anti-p-ERK1/2 (Cell Signaling, #9101, 1:750), anti-ERK 1/2 (Santa Cruz Biotechnology # sc-93, Milan, Italy1:1000), p70 S6 kinase (Cell Signaling, #2708, 1:1000), phospho-p70 S6 kinase (Thr389; Cell Signaling, #9234, 1:1000), Akt (Cell Signaling, #9272, 1:1000), phospho-Akt (Ser473; Cell Signaling, #4060, 1:1000), cofilin (Cell Signaling, #5175, 1:1000) and p-cofilin (phospho Ser3; Abcam, ab12866, 1:1000). After washing, immunoblots were incubated with goat anti-rabbit IgG (GE Healthcare ECL, 1:10000) or anti-mouse IgG antibodies (GE Healthcare ECL, 1:10000) conjugated to horseradish peroxidase (HRP) and visualized on autoradiographic film, using enzyme-linked chemiluminescence (ECL; Immobilion Western, Millipore). The relative protein levels were determined by densitometry and compared with the protein level of the appropriate standard (β-actin for 5-HT7R blots, p70 S6 kinase for phospho-p70 S6 kinase blots, cofilin for p-cofilin blots, and ERK1/2 for p-ERK1/2 blots) probed on the same membrane, after stripping of the antibody previously used. Net intensity value of each band was calculated by subtracting the background of each area from the total intensity.

For 2D-PAGE, monolayer cultures of striatal and cortical cells, treated or untreated with LP-211, were harvested, washed three times with PBS and lysed in the lysis buffer (40 mM Tris-HCl pH 8.0, 8 M urea, 4% CHAPS, 65 mM DTT, and 1 mM PMSF). Proteins were extracted by repeated freezing and thawing with liquid nitrogen and pooled from 3 independent culture dishes. Samples were centrifuged at 17500 g for 15 min at 4°C to eliminate cellular debris and sonicated into an ultrasonic bath for 15 min. Samples were then centrifuged at 17500 × g for 15 min at 4°C. The supernatant was collected and protein concentration determined by the Bradford method, according to manufacturer's instructions (Bio-Rad, Milan, Italy). Lysates were aliquoted and stored at −80°C until use. 50 μ g of lysate proteins were analyzed by 2D-PAGE, as previously described (Speranza et al., 2013). Briefly, samples to be processed by isoelectrofocusing (IEF) were diluted with the rehydration buffer (8 M urea, 0.5% CHAPS, 0.2% DTT, 0.5% IPG ampholytes, and 0.002% bromophenol blue) to a final volume of 125 μ l. The precast IPG strips (3–10 linear pH gradient, 7 cm long, GE Healthcare), used for the first dimension, were passively rehydrated and loaded with the sample at RT for 12 h under low-viscosity paraffin oil. IEF was then performed using an IPGphor isoelectric focusing cell (GE Healthcare), according to the following protocol: 50 V for 3 h, 100 V for 2 h, 500 V for 2 h, 1000 V for 2 h, 3000 V for 2 h, 4000 V for 2 h, 5000 V for 2 h, 6000 V for 2 h, 8000 V until about 25000 V h total. Strips were then equilibrated twice for 15 min under gentle shaking in the equilibration solution (6 M urea, 50 mM Tris-HCl buffer pH 8.8, 30% glycerol, 2% SDS, 0.002% bromophenol blue) containing 1% DTT (to reduce disulfide bonds), in the first equilibration step, and 2.5% iodoacetamide (to alkylate thiols), in the second step. The second-dimension separation was performed on 12% polyacrylamide gels (0.75 mm × 7.5 × 10 cm) by using a Miniprotean II apparatus. The strips were fixed with 0.5% agarose and 0.002% bromophenol blue dissolved in SDS/Tris running buffer. The run was carried out at constant power (10 mA/gel for 15 min; 20 mA/gel until the end of the run). After the second dimension, 2D-gels were electroblotted onto nitrocellulose membranes by using a TransBlot Turbo system (Bio-Rad) following the manufacturer's instructions. The membrane was blocked with 5% milk in TBS-0.1% Tween (TTBS) for 1 h at RT and washed with TTBS. Subsequently, membranes were incubated with primary antibodies diluted in 2.5% milk in TTBS overnight at 4°C. The following antibodies were used: anti-β-actin (BD Transduction Laboratories, #612656, 1:5000); anti-cofilin (Cell Signaling, #5175, 1:1000); β-tubulin (Covance, MMS-435P, 1:1000). Membranes were then washed with TTBS and incubated with the appropriate HRP-conjugated secondary antibody diluted 1:2000 in 2%milk in TTBS for 1 h at RT. Membranes were then washed three times with TBS. Immunoreactive protein bands were visualized by the ECL Plus Western Blotting Detection System (GE Healthcare) according to the manufacturer's instructions.

### Statistical analyses

All the statistical analyses were performed using GraphPad Prism 3.0 (GraphPad Software). Significance of differences was assessed by One Way ANOVA followed by Dunnett *post-hoc* test when drugs-treated cultures were compared to the corresponding control cultures treated with vehicle, or by Tuckey *post-hoc* test for intergroups comparisons. Significance threshold was set at *p* < 0.05.

## Results

### Neuronal cultures from cortex and striatum of mouse brain

**Figure 1:**
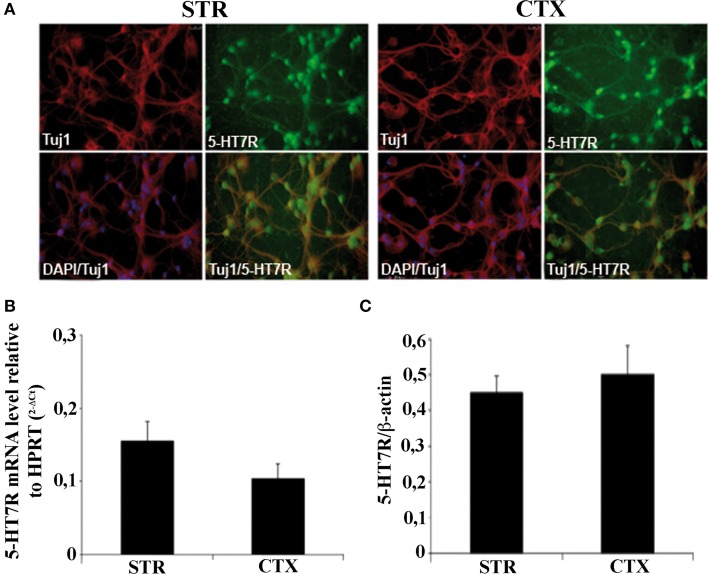
**Characterization of striatal and cortical primary cultures**. **(A)** Photomicrographs of the cells in striatal and cortical cultures immunostained with specific antibodies against neuronal marker Tuj1 (red), and 5-HT7R (green), as indicated in each panel. Cell bodies were counterstained with the nuclear marker DAPI (blue). **(B)** Expression levels of 5-HT7R mRNAs, determined by real time RT-PCR, in neuronal cultures. The bars represent the 5-HT7R mRNA levels normalized with those of the housekeeping gene HPRT (means ± SEM; *n* = 3). **(C)** Quantitation of 5-HT7R protein in neuronal cultures. The bars represent the densitometric values of 5-HT7R Western blot signals normalized with those of β-actin signals in the same samples (mean ± SEM; *n* = 3). STR: cultures from the striatal complex of E15 mouse embryos; CTX: cultures from the cortex of E15 mouse embryos.

As expected from previous data on rat primary cultures (Speranza et al., 2013), the phenotype of cells obtained from the CTX and STR of mouse brains at E15 showed that almost 95% of STR and CTX cells in culture were neurons, as judged by co-localization of Tuj1 antibodies with the nuclear DAPI staining. In addition, about 90% of striatal and cortical neurons were positive to 5-HT7R (Figure 1A).

To evaluate the expression levels of the 5-HT7R in our cultures, we first performed real time quantitative RT-PCR analyses. Although the 5-HT7R mRNAs were slightly higher in the STR than in the CTX, the difference was not statistically significant (Figure 1B). Accordingly, Western blot analyses showed that the abundance of the 5-HT7R protein normalized to that of β-actin, was similar in both areas (Figure 1C).

**Figure 2:**
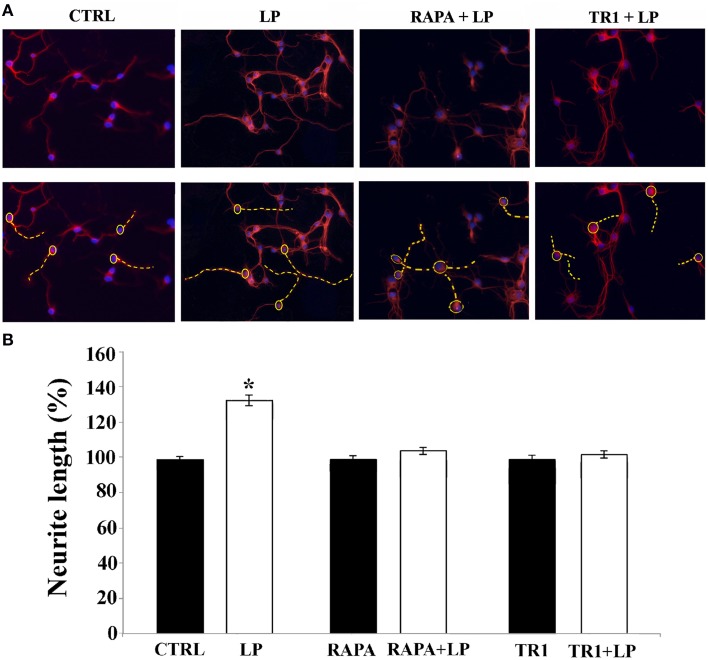
**mTOR signaling pathway is required for neurite elongation induced by 5-HT7R stimulation**. Cortical neurons were treated for 2 h with the 5-HT7R selective agonist LP-211 (LP, 100 nM), or the mTOR inhibitors rapamycin (RAPA, 20 nM), or torin 1 (TR1, 250 nM) with or without LP. **(A)** Representative Tuj1 immunostaining of neuronal cultures (magnification 20x). The images in the lower row are the same of the upper row with the addition of the dashed yellow lines manually drawn by the operator from the soma (yellow circle) to the end of the primary neurite in order to measure neurite length. **(B)** The graph shows the neurite length expressed as percentage of values measured in the corresponding vehicle-treated cultures (CTRL, set to 100%). The bars represent means ± SEM from randomly selected fields for each cell culture condition (*n* = 9). Asterisk (*): value significantly different from CTRL by One Way ANOVA followed by Dunnett *post-hoc* test (*p* < 0.05).

After 3 days in culture, cells were stimulated with the selective 5-HT7R agonist (LP-211; Hedlund et al., 2010) and stained with the Tuj1 antibodies for morphometrical analysis at various time points, in order to measure the neurite elongation. Figure 2A shows representative images of CTRL and LP-211 treated neurons. In line with our previous data (Speranza et al., 2013), we observed that the exposure of neuronal cultures to 100 nM LP-211 caused a significant increase in the length of neurites after 2 h of stimulation in comparison to the control cultures treated with vehicle for the same time length (CTRL, Figure 2B). This effect was specifically due to 5-HT7R, as co-treatment of the cells with LP-211 and the 5-HT7R selective antagonist SB-269970 completely abolished the neurite elongation, while addition of the antagonist alone for 2 h had no effect (data not shown).

### Signal transduction pathways involved in 5-HT7R-dependent neurite elongation

#### mTOR signaling pathway

**Figure 3:**
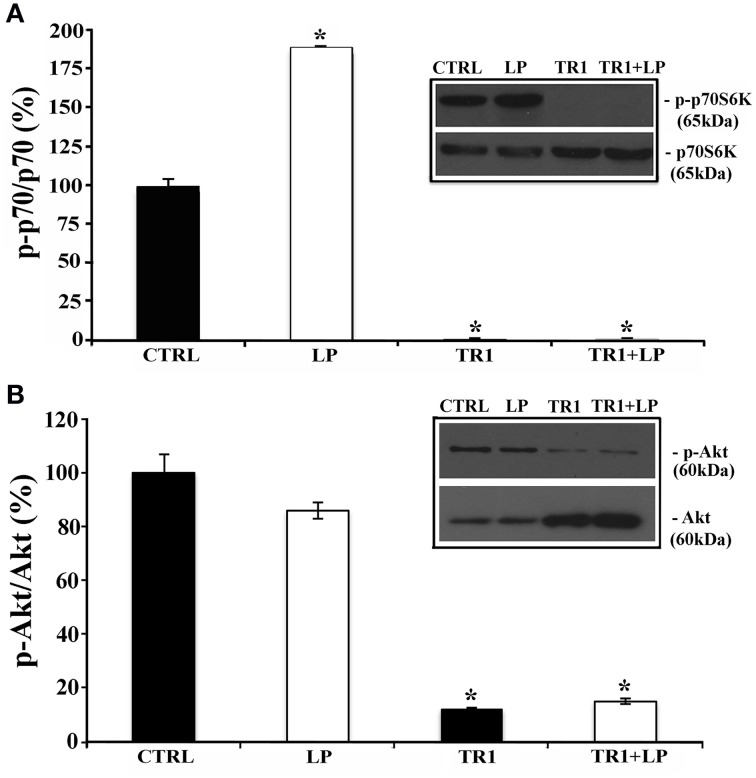
**Stimulation of 5-HT7R activates mTORC1 signaling**. Cortical neurons were treated for 2 h with the 5-HT7R selective agonist LP-211 (LP, 100 nM), or the mTOR inhibitor torin 1 (TR1, 250 nM), alone or in combination. **(A)** The bars show the level of p70S6K phosphorylation at Thr389 (means ± SEM, *n* = 6), expressed as percentage of values measured in the corresponding vehicle-treated cultures (CTRL, set to 100%). The level of p70S6K phosphorylation was measured by Western blot as intensity of p70S6K phosphorylated at Thr389 (p-p70S6K) normalized with that of total (phosphorylated and unphosphorylated) p70S6K in the same samples. The inset displays representative blots probed with antibodies against p-p70S6K and p70S6K; the molecular weights (kDa) are shown on the right. **(B)** The bars show the level of Akt phosphorylation at Ser473 (means± SEM, *n* = 3), expressed as percentage of values measured in the corresponding vehicle-treated cultures (CTRL, set to 100%). The level of Akt phosphorylation was measured by Western blot as intensity of Akt phosphorylated at Ser473 (p-Akt) normalized with that of total Akt (Akt) in the same samples. The inset displays representative blots probed with antibodies against p-Akt and Akt; the molecular weights (kDa) are shown on the right. Asterisk (*): value significantly different from CTRL by One Way ANOVA followed by Dunnett *post-hoc* test (*p* < 0.05).

Since in neuronal cells mTOR signaling plays a pivotal role in dendritic and axonal growth, we chose to investigate its involvement in neurite elongation stimulated by 5-HT7R. To this aim, we used rapamycin, a drug that interacts with and inhibits the mTOR kinase activity, and torin 1, a specific ATP-competitive mTOR inhibitor. As shown in Figure 2B, in cortical cultures the LP-211-induced enhancement of neurite elongation was completely abrogated by the co-treatment with 20 nM rapamycin or 250 nM torin 1. Treatment with rapamycin or torin 1 alone did not affect neurite length. Comparable results were obtained using striatal neurons (data not shown). To further confirm the involvement of the mTOR signaling following agonist stimulation of the 5-HT7R, we investigated the phosphorylation level of the p70 S6 kinase (p70S6K), a direct mTOR downstream substrate (Burnett et al., 1998). To assess the proportion of the phosphorylated form of p70S6K, we performed Western blot analyses using two different antibodies specific for total p70S6K protein (including both phosphorylated and unphosphorylated form) and for the phosphorylated form of the enzyme. The calculated ratio of phospho-p70S6K/ p70S6K was thus used as an index of the mTOR kinase activity. As shown in Figure 3A, we found that treatment of neuronal cultures with LP-211 strongly enhanced p70S6K phosphorylation. Treatment with torin 1, alone or in combination with LP-211, completely blocked this phosphorylation, confirming that it is due to mTOR kinase activity.

mTOR interacts with several proteins to form two distinct complexes named mTOR complex1 (mTORC1) and mTOR complex2 (mTORC2), with different upstream and downstream effectors. mTORC2, but not mTORC1, directly activates the protein kinase Akt by phosphorylating its hydrophobic motif (Ser473; Sarbassov et al., 2005). Therefore, to discriminate between mTORC1 and mTORC2, we analyzed the phosphorylation levels of Akt at Ser473 by Western blot analyses, using two antibodies specific for total Akt protein (including both phosphorylated and unphosphorylated form) and for the phosphorylated form of the enzyme. The phosphorylated Akt isoform, normalized to the total Akt protein did not change significantly in LP-211 stimulated neurons compared to CTRL, suggesting lack of mTORC2 involvement (Figure 3B).

Altogether these results indicate that 5-HT7R-stimulation by LP-211 activates mainly mTORC1 signaling.

#### Cdc42 signaling pathway

**Figure 4:**
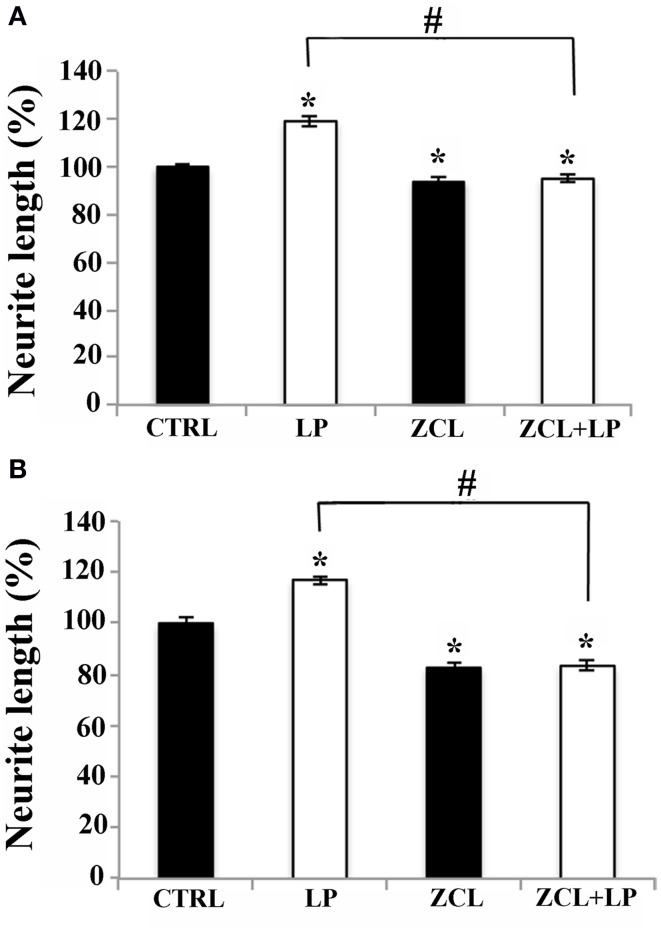
**Cdc42 signaling pathway is required for neurite elongation induced by 5-HT7R stimulation**. **(A)** Striatal and **(B)** cortical neurons were treated for 2 h either with the 5-HT7R selective agonist LP-211 (LP, 100 nM), or with the selective Cdc42 inhibitor ZCL (50 μ M), or with a combination of the two. The graphs show the neurite length expressed as percentage of values measured in the corresponding vehicle-treated cultures (CTRL, set to 100%). The bars represent means ± SEM from randomly selected fields for each cell culture condition (*n* = 6). Asterisk (*): values significantly different from CTRL by One Way ANOVA followed by Dunnett *post-hoc* test (*p* < 0.05). Number sign (#): values significantly different from each other by One Way ANOVA followed by Tuckey *post-hoc* test (*p* < 0.05).

Cdc42 is a protein belonging to the Rho/Rac/Cdc42 GTPase subfamily, that plays a key role in regulating axonal and dendrite morphogenesis (Luo, 2000; Auer et al., 2011). To study its possible implication in neurite outgrowth induced by 5-HT7R stimulation, we treated mouse striatal and cortical cultures with ZCL 278, a selective inhibitor of Cdc42. As shown in Figure 4, the LP-211-induced enhancement of neurite elongation in cultures from both brain regions was completely abolished by the co-treatment with 50 μ M ZLC. It is noteworthy that the treatment with ZCL alone significantly reduced the neurite length in comparison to CTRL of about 10% in striatal cultures and about 20% in cortical cultures. Taken together these data indicate that the Cdc42 signal transduction pathway is involved not only in the enhanced neurite outgrowth induced by LP-211 stimulation of 5-HT7R, but also in time-dependent neurite elongation occurring in basal condition. Thus, this pathway may represent the general signaling system leading to neurite elongation.

#### ERK and Cdk5 signaling pathways

ERK signaling pathway is implicated in neurite outgrowth (Colucci-D'Amato et al., 2003; Jessberger et al., 2009). In addition, we previously demonstrated that in striatal and cortical rat neuronal cultures, the enhanced neurite elongation induced by 5-HT7R stimulation requires ERK phosphorylation and Cdk5 activation (Speranza et al., 2013). Thus, we analyzed the effect of LP-211 on ERK activation also in the striatal mouse neuronal cultures. In line with data on neurite elongation, when neuronal cultures were treated with LP-211 for 2 h, the extent of ERK phosphorylation was significantly increased when compared to CTRL, and this effect was blocked by the co-treatment with 5-HT7R antagonist (SB-269970; Supplementary Figure 1A). These results show that the stimulation of 5-HT7R is responsible for the ERK activation. As expected, the ERK phosphorylation induced by LP-211 was abolished by the treatment with ERK inhibitor, U0126, for 2 h, and U0126 alone also significantly reduced ERK phosphorylation in unstimulated cells (Supplementary Figure 1A). Interestingly, inhibition of ERK phosphorylation by U0126 completely abrogated the enhancement of neurite outgrowth induced by LP-211, while treatment with U0126 alone did not affect neurite elongation (Supplementary Figure 1B).

In line with our previous data (Speranza et al., 2013), we also confirmed that treatment of mouse striatal cultures with roscovitine (inhibitor of Cyclin-dependent protein kinase 5, Cdk5) completely abrogated neurite elongation induced by treatment with LP-211 for 2 h (data not shown).

### Activation of 5-HR7 induces qualitative and quantitative changes of the actin cytoskeleton

**Figure 5:**
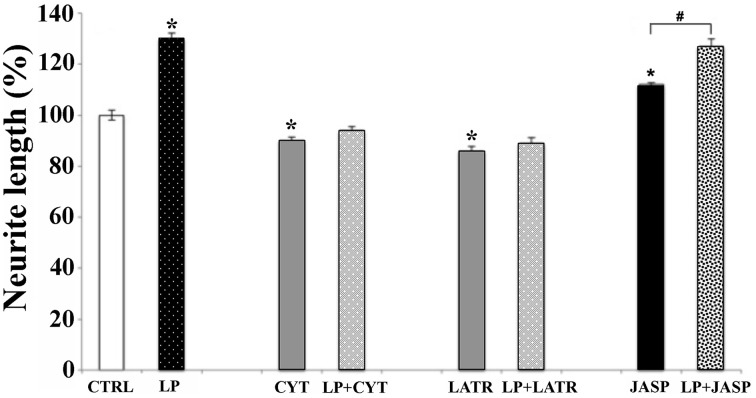
**Actin polymerization is required for neurite elongation induced by 5-HT7R stimulation**. Striatal primary cultures were treated for 2 h with the 5-HT7R selective agonist LP-211 (LP, 100 nM). Cells were also treated with compounds depolymerizing actin filaments, as cytochalasin B (CYT, 100 nM) or latrunculin A (LATR, 2 μM), or promoting actin polymerization as jasplakinolide (JASP, 2 μM), with or without LP. The graph shows the neurite length expressed as percent values in reference to vehicle-treated cultures (CTRL, set to 100%). The bars represent means ± SEM from randomly selected fields for each cell culture condition (*n* = 9). Asterisk (*): values significantly different from CTRL by One Way ANOVA followed by Dunnett *post-hoc* test (*p* < 0.05). Number sign (#): values significantly different from each other by One Way ANOVA followed by Tuckey *post-hoc* test (*p* < 0.05).

To establish the relationship between 5-HT7R activation and actin cytoskeleton dynamics, we treated mouse striatal cultures with 100 nM cytochalasin B or 2 μM latrunculin A. These agents, promoting net depolymerization of the actin filaments, nearly abolished the neurite outgrowth stimulated by LP-211, indicating that this enhanced elongation required the polymerization of actin pool (Figure 5). When added to the culture media without LP-211, these two compounds significantly reduced the neurites length in comparison with the vehicle-treated cells (CTRL). Accordingly, when the striatal neurons were exposed to the agent promoting actin polymerization (jasplakinolide, 2 μM) the length of their neurites significantly increased (Figure 5). Interestingly, when neurons were co-treated with jasplakinolide and LP-211, the effect was not cumulative and the neurites length was similar to the one obtained with LP-211 alone. Collectively, these results indicate that the neurite outgrowth induced by LP-211 depends on modulation of actin cytoskeleton dynamics.

**Figure 6:**
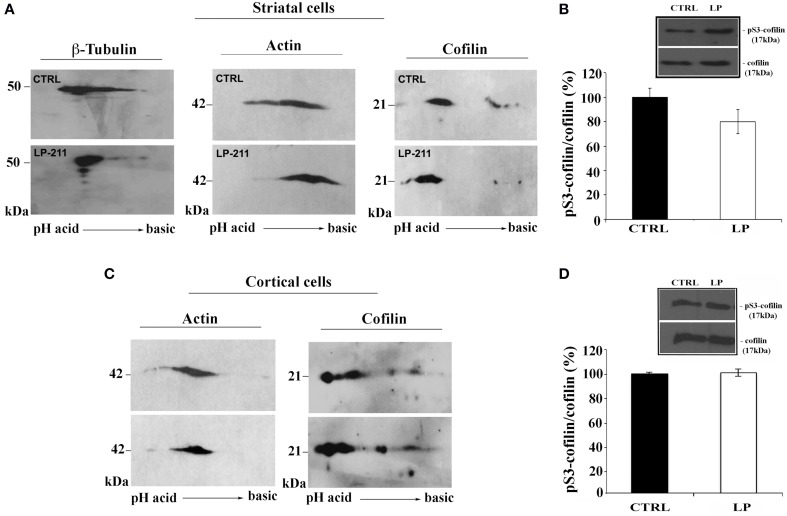
**Effect of LP-211 on expression levels of several cytoskeletal proteins in neuronal primary cultures**. 2D immunoblot analyses, using antibodies against β-tubulin, actin or cofilin, were performed on **(A)** striatal cells or **(C)** cortical cells treated with vehicle (CTRL) or with the 5-HT7R selective agonist LP-211. The graphs on the right show the level of cofilin phosphorylation on Ser 3 (pS3-cofilin) in striatal **(B)** and cortical **(D)** neurons treated for 2 h with LP-211 (LP, 100 nM). The intensity of pS3-cofilin was normalized with that of total cofilin (means ± SEM; *n* = 3) and was expressed as percentage of values measured in the corresponding vehicle-treated cultures (CTRL, set to 100%). The insets display representative SDS-PAGE blots probed with antibodies against pS3-cofilin and cofilin. The molecular weights (kDa) are indicated on the right.

To further investigate the role of the cytoskeleton in LP-211-induced neurite elongation, we have studied the effects of LP-211 on mouse striatal and cortical cultures using 2D protein analyses of β-tubulin, β-actin and cofilin. Immunostaining of 2D-gels of both control striatal and cortical cultures showed a marked stretching of both β-tubulin and actin spots, attesting a high level of protein heterogeneity in terms of post-translational modification. These protein stretches were not substantially affected upon treatment with LP-211 with the exception of a reduction of the actin acidic spots, more marked in striatal neurons (Figures 6A,C). Interestingly, LP-211 treatment was found to alter the 2D expression profile of cofilin, a regulator of actin filament assembly/disassembly, in striatal and cortical cells. Indeed, a shift of the stretch of cofilin spots at 21 kDa toward acidic pH region was observed following LP-211 treatment.

SDS-PAGE and Western blot analyses using antibodies for total cofilin and for cofilin phosphorylated at Ser3 (pS3) did not show significant difference between CTRL and LP-211 treated striatal or cortical neurons (Figures 6B,D). These results indicate that the shift of cofilin toward the acidic region observed in 2D-gels following stimulation of the 5-HT7R cannot be attributed to its phosphorylation at Ser3.

### Activation of 5-HT7R stimulates axonal outgrowth

**Figure 7:**
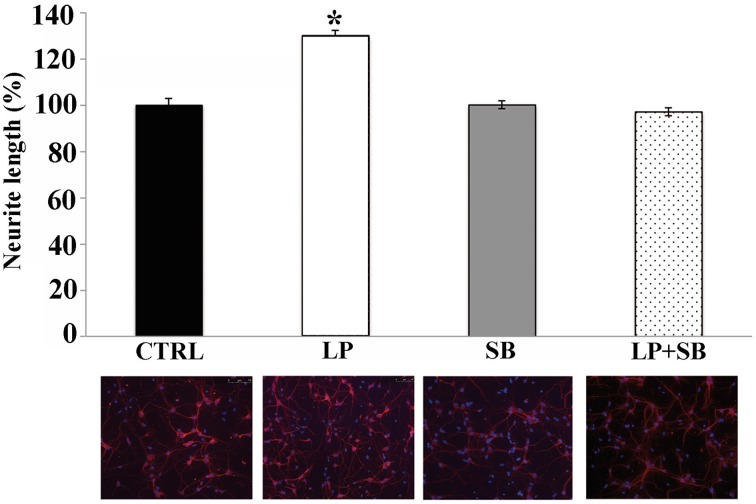
**Stimulation of 5-HT7R enhances neurite outgrowth in hippocampal primary cultures**. Hippocampal cells were treated for 2 h with the 5-HT7R selective agonist LP-211 (LP, 100 nM), alone or in combination with the selective 5-HT7R antagonist SB269970 (SB, 100 nM). Neurite length was measured on cells stained with anti-Tuj1 antibody, and expressed as percentage of values measured in the corresponding vehicle-treated cultures (CTRL, set to 100%). The bars represent means ± SEM from randomly selected fields for each cell culture conditions (*n* = 10). Asterisk (*): values significantly different from CTRL by One Way ANOVA followed by Dunnett *post-hoc* test (*p* < 0.05). The panels below each bar display representative images of hippocampal neurons immunostained with the neuronal marker Tuj1 (red) and counterstained with the nuclear marker DAPI (blue; magnification 20x).

Although neurite outgrowth is studied in cultured neurons before the occurrence of neuronal polarization, it is generally considered the equivalent of axonal extension (Conti et al., 1997). To determine whether 5-HT7R could indeed modulate axonal growth, we used microfluidic culture platforms that allow physical separation between soma and axons of cultured neurons (Park et al., 2006). Microfluidic chambers have been mostly used with HIPP neurons for their ability to develop long axons. Therefore, we first tested the effect of 2 h stimulation with LP-211 on neurite elongation of E18 mouse HIPP neurons cultured in traditional chamber slides. These cultures were immunostained with TuJ1 antibodies and counterstained with the nuclear marker DAPI, indicating that most of cultured cells were neurons (see panels in Figure 7). Consistent with previous results obtained with other 5-HT7R agonists (Kvachnina et al., 2005), we observed that the length of neurites significantly increased in LP-211-treated cultures compared to CTRL; the co-treatment of neurons with LP-211 and SB-269970 completely abolished neurite elongation, while addition of the antagonist alone did not affect neurite outgrowth (Figure 7).

**Figure 8:**
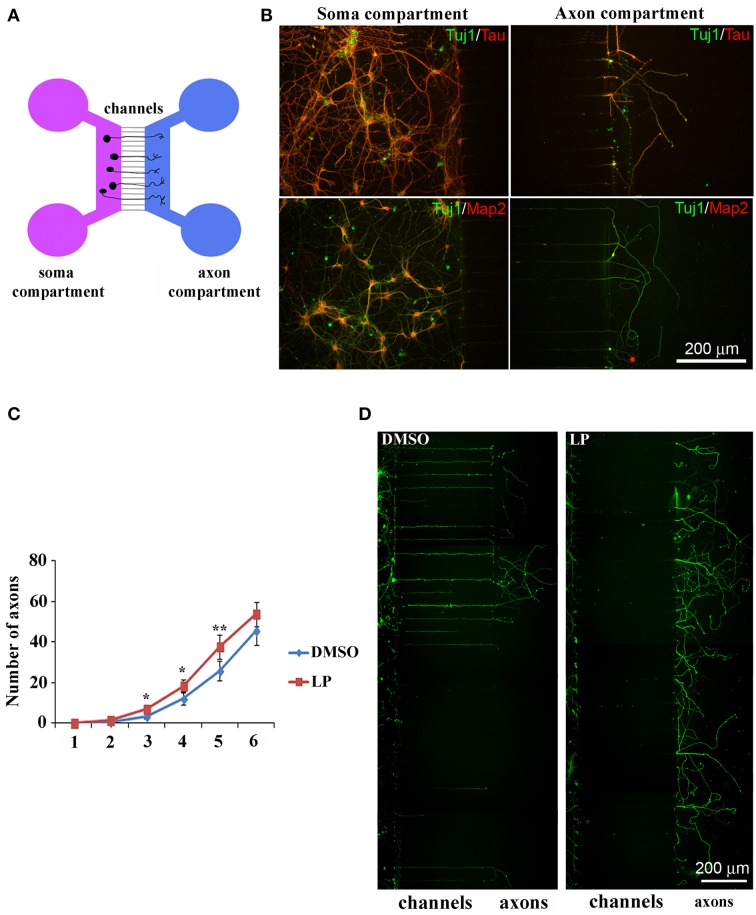
**Stimulation of 5-HT7R enhances axonal outgrowth in cultured hippocampal neurons. (A)** Schematic representation of microfluidic chambers. **(B)** Co-immunolabeling of neurons cultured in microfluidic chambers with the neuronal marker Tuj1 (green) and either the axonal marker Tau (top row, red) or the dendritic marker Map2 (bottom row, red). Dendritic immunolabeling is confined in the soma compartment and only axons cross the micro-channels to reach the other compartment. **(C)** The number of axons crossing the micro-channels is significantly higher in LP-211 treated cultures (LP, red line), respect to control (DMSO, blue line), after 3, 4, and 5 DIV. Asterisk (*): values significantly different from DMSO by One Way ANOVA followed by Dunnett *post-hoc* test (*p* < 0.05). **(D)** Representative hippocampal cultures grown in the presence of DMSO or LP and immunolabeled with the Tuj1 antibody (green).

Then, we cultured HIPP neurons in microfluidic chambers (Park et al., 2006), implementing culture medium with 100 nM of the agonist. Neurons were plated on one side of the culture chamber (soma compartment) and axons, but not dendrites, grew into the other compartment, passing through interconnecting micro-channels (450 μm long) (Figure 8A). We first verified whether in our system the only fibers crossing the channels were axons, by co-immunolabeling hippocampal neurons with Tuj1 (neuronal marker) and either anti-Tau (axon specific marker) or anti-Map2 (dendrite specific marker). As expected, the anti-Tau antibody labeled axons in both compartments, while dendritic labeling with Map2 was confined to the soma compartment (Figure 8B). Therefore, we plated HIPP neurons in the presence of either LP-211 or vehicle (CTRL), added to both compartments. Cultures were maintained for 6 days and axons reaching the appropriate compartment were counted daily, from 1DIV to 6DIV. In LP-211-treated cultures we observed a significant increase in the number of axons crossing the channels by 3 to 5DIV, clearly indicating that stimulation of the 5-HT7R promotes axonal growth, at least at early stages (Figure 8C). This difference disappeared at 6DIV, suggesting receptor desensitization, although we have also considered the hypothesis that the number of axons would eventually reach a plateau, since the number of channels to cross is limited. Figure 8D shows two typical cultures, grown in the presence of either DMSO or LP-211, indicating that, before plateau is reached, the number of axons reaching the side compartment is higher in the presence of 5-HT7R agonist.

## Discussion

Neurotransmitters have a well-established role for neuronal communication in the adult CNS, but many of them, including serotonin, have been shown to act as signaling molecules during neuronal development regulating neurite outgrowth, dendritic spines shape and number, target selection and synapse formation (Van Kesteren and Spencer, 2003; Lesch and Waider, 2012).

Accordingly, a role for 5-HT7R in the regulation of neurite outgrowth was demonstrated on mouse hippocampal neurons *in vitro* (Kvachnina et al., 2005), and more recently on rat striatal and cortical cultures (Leo et al., 2009; Speranza et al., 2013). In line with these findings, in this paper we demonstrate that the neurite outgrowth observed in cultured embryonic neurons of mouse striatum, cortex and hippocampus is strongly stimulated by LP-211, a newly discovered highly selective 5-HT7R agonist (Hedlund et al., 2010). We show that this process requires the activation of the serine/threonine kinase mTOR, and the Rho GTPase Cdc42, in addition to Cdk5 activation and ERK phosphorylation, corroborating and extending our previous findings on rat neuronal cultures (Speranza et al., 2013).

In neuronal cells, mTOR is implicated in multiple processes, including transcription, ubiquitin-dependent proteolysis, and microtubule and actin dynamics, all of which are crucial for neuronal development and long-term modification of synaptic strength (Jaworski and Sheng, 2006). Moreover, mTOR is recruited following activation of various G protein-coupled receptors and thereby plays a role in synaptic plasticity through formation and maturation of new synapses (Hoeffer and Klann, 2010; Meffre et al., 2012). The two distinct multiprotein complexes formed by mTOR with different accessory proteins, mTORC1 and mTORC2, are differently sensitive to rapamycin (Takei and Nawa, 2014). Recent efforts have led to the development of torin 1, an ATP-competitive mTOR inhibitor blocking both mTORC1 and mTORC2 complex activity (Liu et al., 2010). Our results show for the first time that the neurite elongation induced by agonist stimulation of 5-HT7R is dependent on mTOR, as the outgrowth is completely inhibited by both rapamycin and torin 1. Accordingly, phosphorylation of the p70SK, one of the well known targets of mTOR (Laplante and Sabatini, 2012), was strongly increased by treatment of neuronal cultures with LP-211, and completely abolished by co-treatment with torin 1. These results are in agreement with the recent findings that the *in vivo* treatment with LP-211 stimulates mTOR signaling in the mouse brain (De Filippis et al., 2014) and highlight the key role of this pathway in mediating the effects induced by 5-HT7R stimulation in the CNS. On the other hand, our analyses indicate that phosphorylation of Akt, one of the well known targets of mTORC2 (Takei and Nawa, 2014), was not affected by the treatment of neuronal cultures with LP-211, suggesting that the intracellular cascade stimulated by 5-HT7R involves mainly mTORC1.

Another piece of the puzzle was provided by the recent studies in which Rho GTPases have been identified as key regulators of actin cytoskeleton rearrangement leading to axonal and dendritic growth. Thus, it has been suggested that Rho GTPases may act as a molecular switches integrating signals from the extracellular environment (Auer et al., 2011; Li et al., 2014). As a member of Rho GTPases protein family, Cdc42 is involved in neuronal morphogenesis including axon growth and guidance, dendritic spine plasticity and synapse formation (Luo, 2000). This GTPase is required for filopodia formation and for the concomitant activation of the actin regulator cofilin in growth cones (Garvalov et al., 2007). In accordance with these observations, our results clearly indicate that Cdc42 indeed plays a crucial role in the LP-211-dependent enhancement of neurite outgrowth. Thus, this Rho GTPase may be considered a key molecule in the signaling pathways coupling activation of 5-HT7R with the cytoskeleton reorganization.

As aforementioned, we previously demonstrated that ERK and Cdk5 are also activated during the LP-211-induced enhancement of neurite outgrowth (Speranza et al., 2013), which has been corroborated here in cultured neurons obtained from various regions of the mouse brain (Supplementary Figure 1). The involvement of ERK and Cdk5 are consistent with the well-known role of these pathways as critical signal transmission hubs positioned between a range of cell surface receptors and various cytoskeletal systems regulating neuronal morphology (Colucci-D'Amato et al., 2003; Jessberger et al., 2009). Thus, our new experimental evidence showing that mTOR and Cdc42 are also involved in the signaling pathways activated by 5-HT7R suggest that this newly found member of the 5-HT receptor family uses a variety of molecular tools to exert its effect on neurite outgrowth during development. Accordingly the link between some of these signal trasduction pathways has been demonstrated in several different experimental systems (Kvachnina et al., 2005; He et al., 2013). A plausible hierarchical order for these downstream 5-HT7R signaling components would envisage recruitment of Cdk5 and Cdc42, in analogy to recent findings by Duhr et al. (2014). These authors have shown that both these signaling pathways are involved in the 5-HT6R neurite elongation, and that Cdk5 constitutively interacts and phosphorylates the receptor. This cooperation might occur also for 5-HT7R, and Cdk5 would in turn activate ERK signaling, as observed in rat primary cultures (Speranza et al., 2013). ERK pathway might then activate mTORC1, as reviewed by Laplante and Sabatini (2012). In addition, Cdc42 could be a downstream Cdk5 mediator of neurite growth, operating in parallel to ERK pathway (Cheung et al., 2007).

The protruding force responsible for the extension of neuronal processes relies on the modulation of cytoskeleton dynamics, in particular on the regulation of microtubules and microfilaments polymerization (Schaefer et al., 2008). Accordingly, our data indicate that the neurite outgrowth induced by 5-HT7R stimulation depends on actin filaments. Indeed the stimulating effect of LP-211 on neurite outgrowth was blocked by the addition of actin-binding drugs that promote net depolymerization of actin filaments, either by sequestering the free actin monomer pool (latrunculin) or by capping the fast-growing barbed ends of actin filaments (cytochalasin). On the other hand, the treatment with jasplakinolide, which stabilizes polymerization of actin filaments, increased the neurite length in the absence of LP-211. However, the co-treatment with jasplakinolide and LP-211 did not promote additional neurite outgrowth compared to the treatment with the agonist alone, suggesting that 5-HT7R activation may have stimulated the outgrowing mechanism at its maximum level. It is important to consider that the modulation of actin dynamics is an extremely complex process, as revealed by either stimulatory or inhibitory effects of actin polymerization drugs on axonal or dendritic growth (Bradke and Dotti, 1999; Gallo et al., 2002; Jones et al., 2004). These apparently divergent results may be attributed to the different stages of development/differentiation of neuronal processes, as well as the various experimental conditions (i.e., drug treatment, neuronal cultures) used by different investigators. Altogether, our data are consistent with the knowledge that the cellular mechanisms regulating neurite outgrowth are deeply influenced by actin dynamics (Schaefer et al., 2008).

Many extracellular signaling molecules enhancing neurite outgrowth exert profound effects on the organization of the actin cytoskeleton, mainly acting on actin-binding proteins, such as cofilin. The regulation of cofilin phosphorylation is a key convergence point of multiple cell signaling networks that link extracellular stimuli to actin cytoskeletal dynamics (Huang et al., 2006, 2013; Mizuno, 2013). It has been suggested that cofilin activity, modulated by a proper balance of its phosphorylation level, may act as a regulator for neurite extension in specific subcellular regions of the neuron (Endo et al., 2007; Figge et al., 2012). Interestingly, recent data indicate that stimulation of 5-HT7R is able to reverse the abnormal activation of cofilin in a mouse model of Rett syndrome (De Filippis et al., 2014). Here, following 5-HT7R agonist stimulation of both striatal and cortical neurons, we didn't detect significant changes of cofilin phosphorylation at Ser3 by Western blot analyses. On the other hand, the 2D gel profiles of cofilin protein from LP-211 treated cultures undergo significant changes compared to control cultures, showing a shift toward acidic pH of multiple spots without changes in molecular mass. These changes may be ascribed to phosphorylation on residues different from Ser3, or to other post-translational modifications. The detailed analyses of these modifications will be object of further investigation.

Many experiments using cultured embryonic hippocampal neurons have revealed that, as they develop, neurons initially generate several equivalent neurites, but then begin to polarize so that one neurite becomes an axon while the remaining neurites become dendrites (Dotti et al., 1988).

Interestingly, here we demonstrate for the first time that stimulation of the 5-HT7R enhances axonal elongation in cultured hippocampal neurons. These data, coupled with previous results indicating involvement of 5-HT7R in potentiating formation of dendritic spines in hippocampal neurons, strongly support the emerging role of 5-HT7R in shaping brain networks during development by modulating neuronal cytoarchitecture and connectivity (Kobe et al., 2012; Volpicelli et al., 2014).

In addition, the stimulating effect of 5-HT7R on axonal elongation open a new perspective suggesting the involvement of 5-HT7R in axonal pathfinding and regeneration, presumably through the activation of mTOR signaling and cofilin. Indeed, recent data indicate that mTOR pathways and cofilin activation play a key role in the mechanism underlying the ability of axon to regenerate (Stern et al., 2013; Lu et al., 2014).

Interestingly, the successful rescue of functional and behavioral deficits observed in animal models of Fragile X syndrome and Rett syndrome (Costa et al., 2012; De Filippis et al., 2014), following stimulation of the 5-HT7R, identify this receptor as a potential target for innovative pharmacological treatment of several neurodevelopmental diseases associated with abnormal CNS connectivity.

In summary, our results highlight the role of 5-HT7R and its signal transduction pathways in shaping neuronal morphology in embryonic forebrain neurons in culture, suggesting its involvement in the correct establishment of neuronal wiring during critical periods of CNS development. Similar mechanisms could operate also in the mature brain to modulate plasticity of neural circuits.

## Author contributions

LS, TG, MEDS, LL, AC, and EL performed experiments; FV, GCB, and ML analyzed data; UdP, MC, and CP-C designed research and wrote the paper.

### Conflict of interest statement

The authors declare that the research was conducted in the absence of any commercial or financial relationships that could be construed as a potential conflict of interest.

## Abbreviations

5-HT: 5-hydroxytryptamine
5-HT7R: serotonin receptor 7
Cdc42: cell division cycle 42
Cdk5: cyclin-dependent kinase 5
CTX: cortex
E: embryonic age
ERK: extracellular signal-regulated kinases 1/2
HIPP: hippocampus
mTOR: mammalian target of rapamycin
p70S6K: p70 S6 kinase
STR: striatal complex.
