# Supplementary material for: Activation of 5-HT7 receptor stimulates neurite elongation through mTOR, Cdc42 and actin filaments dynamics
Source: Front Behav Neurosci. 2015 Mar 11;9:62. doi: 10.3389/fnbeh.2015.00062 (PMC4356071; doi:10.3389/fnbeh.2015.00062)
Supplement: Supplementary file 1 [file Image1.PDF]

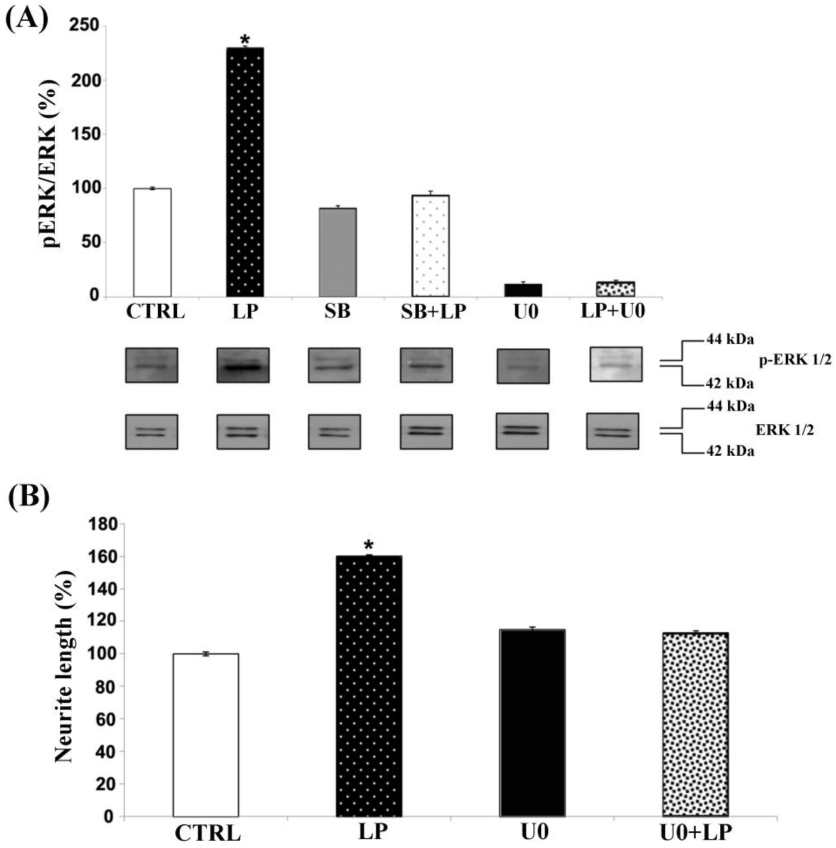

Supplementary Figure 1: Neurite elongation induced by 5-HT7R stimulation requires activation of ERK signaling pathways. (A) Striatal neurons were treated for 2 h either with the selective 5-HT7R agonist LP-211 (LP, 100 nM) alone, or in combination with the selective 5-HT7R antagonist SB269970 (SB, 100 nM). Cells were also treated with the selective ERK inhibitor U0126 (U0, 10 µM) with or without LP-211. The level of ERK phosphorylation was measured as intensity of phosporylated ERK (p-ERK 1/2) normalized with that of total ERK (ERK 1/2) in the same samples. ERK phosphorylation (means ± SEM; n=6) was expressed as percentage of values measured in the corresponding vehicle-treated cultures (CTRL, set to 100%). The boxes below each graph display representative blots probed with antibodies against p-ERK 1/2 and ERK 1/2. The molecular weights (kDa) are indicated on the right. **(B)** Striatal cells were treated for 2 h either with the selective 5-HT7R agonist LP-211 (LP, 100 nM) alone, or in combination with U0126 (U0, 10 µM). The graph shows the neurite length expressed as percentage of values measured in the corresponding vehicle-treated cultures (CTRL, set to 100%). The bars represent means ± SEM from randomly selected fields for each cell culture condition (n=10). Asterisk (*): values significantly different from CTRL by one way ANOVA followed by Dunnett post-hoc test ($p < 0.05$).
